# Abstract of the Proceedings of the American Association of Obstetricians and Gynecologists at the First Annual Meeting

**Published:** 1888-10

**Authors:** 


### *ABSTRACT OF THE PROCEEDINGS OF THE AMERICAN ASSOCIATION OF OBSTETRICIANS AND GYNECOLOGISTS AT THE FIRST ANNUAL MEETING,*

HELD IN WASHINGTON, D. C., SEPTEMBER 18, 19 AND 20, 1888.

### FIRST DAY—MORNING SESSION.

The President, DR. W. H. TAYLOR, of Cincinnati, called the meeting to order, and introduced DR. THOMAS E. MCARDLE, of Washington, who, in the name of the medical profession of Washington, cordially welcomed the association to the city, and congratulated the members upon their organization for the performance of the important work they had undertaken.

The PRESIDENT then delivered his annual address, a brief abstract of which is as follows:

In thus assembling for the first time as a Society, it may be proper to demand a reason for its existence. The daily round of professional experience impresses the practitioner of medicine with the incompleteness of his knowledge, and to the man who sees in his avocation something more than a mere source of pecuniary profit there must arise the desire to know the yet unknown. No argument is needed to prove the assertion that the united effort of many is more fruitful than the inharmonious working of individuals; hence the propriety of cooperative organization. It may be admitted that the motive of our Association is, first, our own advancement, yet it cannot be considered undue self-adulation if we believe that from such combined effort good must come to the profession at large, and necessarily through the profession to those who, above all others, are interested in the perfection of our knowledge and skill—our clients.

The activity of the past decade has given us so many important facts, that it would scarcely be hyperbole to say that a new practice of obstetrics and gynecology has been created in that time. But the very fact that there has been such progress, and that so much that but a few years ago was impracticable or even unknown is now feasible and well known, only stimulates us to better work and further advance; and, with the vast range of study and diversity of subjects now comprehended by the science of medicine, it is clear, beyond controversy, that advance can be made only by men directing their efforts to a limited field of work. Such, gentlemen, I believe, are sufficient reasons for our presence here to-day as the American Association of Obstetricians and Gynecologists.

While I have felt the high honor of my position, I have also realized the great difficulties of bringing anything before you worthy of your attention. A glance at the programme laid before you will convince you that the various practical topics which are so important are to constitute much of the subject matter of discussion here, and, therefore, it is inexpedient for me to dwell upon them. The proper indications for abdominal section, the true position which electricity shall occupy in gynecology, the propriety of hysterectomy, the value of Alexander's operation, and its newly-devised congener, abdominal fixation for retrodeviations of the uterus, the relative merits of craniotomy, Cesarean section and induced labor, the best method of dealing with extra-uterine pregnancy, the proper means of securing antisepsis, are all questions fraught with great interest, and merit the most thorough study; and, so confident am I that, before this meeting closes, much light will be shed upon many or all of them by our deliberations, I shall pass them by without further consideration.

An opinion has large credence among the laity, and is endorsed by some members of our profession, that intentional abortion is a common crime, while statisticians and social scientists see in the decreasing fecundity of American women clear evidence of the truth of this charge. That some women are guilty, admits of no doubt, but that it is so universal an evil as sensational literature, especially of non-professional origin would indicate, I do not believe. Permit me to direct your attention to a more demonstrable cause for diminishing fertility. I need but remind you of the deleterious influence of social habits and circumstances over the functions and physique of women, and, consequently, how many fashionably-educated girls are incapable of completing the process of maternity. Those who are fond of charging American women with the crime referred to cite to us the rich, the educated, the refined, as guilty, while their sisters who are less favored financially, and are humbler socially, bear their due proportion of children. Now, the first class are the physically feeble, the enervated, and, therefore, incapable of carrying the process of reproduction to its ultimate perfection. A lack of fecundity on the part of a woman is often due to endometritis, and is overcome by the cure of the diseased condition. Many cases of sterility after the birth of one child are due to inflammatory processes of the genitalia. Accept these statements as true, and a rational explanation of the asserted evil is found, which at once transfers it from the domain of ethics and morals to within the pale of our professional care, and at the same time vindicates those whom we look upon as the purest and best, from the indiscriminate charge of the murder of their unborn children.

I believe the influence of the genitalia over the general system, especially in its nervous manifestations, has been exaggerated; that entirely too much attention has been paid to the genital organs in connection with the neuroses. Our associate, Dr. W. H. Wathen, has recently said: "The removal of healthy ovaries or tubes to cure epilepsy or vague nervous diseases not due to irritation of the pelvic organs, was not more consistent than the castration of a man for similar purposes." And our associate Dr. A. Vander Veer's experience regarding hystero-epilepsy was that it had not been cured by removal of the appendages.

A subject of practical importance urgently demanding attention from the obstetrical section of our association is the constantly-increasing inability of women, who should be "nursing mothers," to furnish milk for their infants. Many women who have borne children are incapable of nourishing them; probably about forty per cent. with us, and, according to Escherich and others, in Bavaria, fifty-nine per cent. of mothers become incapable from physical causes of nursing their children within two months after delivery. In a large degree this is the result of habits tending to impair the integrity of the mammary glands.

Another topic well worthy of our most careful thought is, what can be done to mitigate the pains of labor? My impression is that, with the great majority of obstetricians, but little is done to lessen pain during a large part of the process of delivery. With the abundant therapeutic resources of the present day, we ought surely to divest this ordeal of the intensity of its pain. I trust by another meeting to report upon experiments I am now making.

It has been said, as civilization progresses, the tendencies of all human diseases has been to assume the neurotic type, and also that diseases become more complex in their manifestation. On the other hand, the recent advances in antisepsis allow the hope that some day we may eradicate the septic diseases of child-birth, and that our grand advances in surgery shall qualify us to cope successfully with what a few years ago would have been considered a helpless and hopeless condition. Science knows no sentiment, and we can cherish the achievements of the past only so far as they aid us for the future. Science almost every day brings a new surprise, so we may confidently anticipate advances and successes which shall as far surpass our present attainment as these do those of the past generation, and happy may we count ourselves that this association, so auspicicusly established, shall contribute to these magnificent and beneficent attainments.

Dr. A. Cordes, of Geneva, Switzerland, sent two memoirs, which were read by the Secretary, Dr. W. W. Potter. The first paper, on the

TREATMENT OF PUERPERAL ECLAMPSIA, contained a report of four cases treated by the injection of bromide of potassium and chloral hydrate, with abstraction of blood over the mastoid processes by leeches; advocating this treatment in preference to general bleeding, chloroform, and arterial sedatives. He also deprecated any interference with the genitala, such as digital examination, passing the catheter, etc., which he thought were liable to provoke convulsions.

The second paper,

THE TREATMENT OF ENDOMETRITIS

by injections of pure nitric acid was illustrated by three cases in which this method was employed. The writer preferred this method of treatment to that by the curette, in cases where there were fungosities or other irregularities of the intra-uterine surfaces.

Dr. A. Lapthorn Smith (Montreal).—I would like to be permitted to say two or three words on this very important subject. I think we should lay down three principles to guide us in these cases. Puerperal convulsions are due to mechanical pressure on the renal veins. If that remains too long, it will bring on uremia. Uremia continued long enough produces permanent damage to the brain. We should empty the uterus and take the pressure off the renal veins at the earliest moment we are sure such a condition is in existence. I have acted myself two or three years on that plan, and have had no cause to regret it. The only regret I have is that during five or six years I adopted the method of waiting. Although none of the patients died, all of the children died, and one of the patients was in an asylum several years. I think if premature delivery is brought on under antiseptic precautions, we will in all cases save the woman, and save her brain intact, and occasionally save the child. But the child is a secondary consideration.

Dr. Byron Stanton (Cincinnati).—I would like to ask if bringing on labor is not adding fuel to the flame; if it is not better to defer as long as possible the induction of labor, and treat the renal condition; place the patient in better condition for delivery. We know that convulsions do not always cease at delivery.

Dr. A. L. Smith.—I do not think you should wait until the woman has convulsions. If you know that the urine is loaded with albumin, bring on labor without delay. Every moment of delay you increase the danger.

Dr. Joseph Price (Philadelphia).—The gentleman that has just taken his seat expresses a great deal of wisdom in his remarks. I will simply state one case out of quite a number in my own experience. Mr. Tait calls attention to the frequency of such troubles in elderly primiparæ. It is claimed that it is exceedingly common in that class of patients. I will merely cite one case, to fortify the remarks of the gentleman from Montreal. I was summoned to Germantown to see a patient 30 years of age, married two years. At the seventh month of pregnancy, she was enjoying excellent health, and was watched by a very careful practitioner. There was no albumin in the urine, or other indication of approaching mischief. At the eighth month she came to him with edematous feet, headaches, puffy face, and disturbed vision. He wrote me he would like me to see a patient; that she was pregnant, and he feared convulsions. Shortly after, he telegraphed me that she had had a convulsion, and to come on the first train. She had had two convulsions. Her urine, secured only by the catheter, was found to be solid with albumin. In the interest of the mother and child we agreed upon, with some preparation, induced labor. This preparation, it strikes me, is of paramount importance in the majority of these cases of approaching eclampsia. We agreed upon the use of a hydragogue cathartic and the administration of bromide and chloral. Her brain and blood, and particularly her kidneys, were relieved of this puerperal poison. Her physician applied the forceps, and delivered the child. Her kidneys cleared up rapidly, no further brain symptoms appeared, the edema disappeared, and he saved both mother and child. I tell this as simply a typical case. I could cite a number of others. I wish to emphasize the importance of this careful preparation and early induction of premature labor in cases due to mechanical pressure.

Dr. W. W. Potter.—I had not thought that I should say a word on this subject, but Dr. Price has kindly opened up a channel of thought which is agreeable to me, because he refers to a method that has brought success in my hands. I will very briefly rehearse a recent case. On the fourth of June last, I was called to see a woman who had been in almost a uremic stupor several days; not absolutely comatose, but verging toward it. She was then about the end of the eighth month of pregnancy. I saw her, and induced labor. I delivered her on the morning of the fifth. She was unconscious of her maternity for some time. The child weighed from three and one-half to four pounds. For two weeks she was in only a semi-conscious condition. She was edematous to an extreme degree. She has fully recovered, and the child is living, but she never had a drop of milk from the first. She was a well-developed woman without any appearance of ill-health. It was something like a month before she was really well so that she could go out. I mention this as a parallel to the remarks of Dr. Price. We preceded the work with an injection of a grain of morphia hypodermatically.

Dr. Clarke (Cambridge).—I consider that emptying the uterus is one important thing in eclampsia. Some three years since, I was called in consultatation to see a woman in convulsions, about the seventh month of pregnancy. We decided that it was necessary to dilate the uterus and deliver the woman. Previous to this we used the subcutaneous injection of morphia, one-half grain, which was repeated some time after the woman was delivered. What seemed to relieve her a great deal was one-sixteenth of a grain of pilocarpine. It was a long time before she recovered her sight. The next time she was pregnant I commenced treatment early, using saline laxatives. She went on well with scarcely any albumin.

THE INDICATIONS FOR DRAINAGE IN ABDOMINAL SURGERY.

By Dr. Joseph Price, of Philadelphia.—He said the object of this paper was to point out the indications for drainage in abdominal surgery. At the present time, there is the greatest variance in the opinions and practice of surgeons upon this point, and some fixed rules, such as are laid down in other departments of operative surgery, are needed.

First: Is there danger from the retention of fluids in the periton eal cavity? Second: Is the drainage-tube a safe means of obviating this danger? Third: If the tube involves danger, what are the comparative risks from its employment or omission? That the peritoneum will relieve itself of exuded fluids is well known; and it is also an established fact that the free use of saline cathartics will often arrest acute peritonitis. Experience has also shown that these resources often fail, and it is necessary to reopen the abdomen, irrigate the peritoneum, and insert a tube; this, too, in cases where no pus is found. It is a fallacy to believe that extensive adhesions are alone an essential indication for drainage, since free exudation can arise from severing slight adhesions, and extensive bleeding follow slight oozing after the patient reacts. This latter fact was illustrated in a recent case in which bloody exudation, which seemed almost nothing at the close of the operation, flowed freely through the tube for ten days afterward. A great difficulty is presented in deciding a case to be simple, particularly in cases of inflammation of the tubes and ovaries. Indeed, no one can decide during an operation whether the inflammation is simple, purulent, or specific. Herein is an additional indication for the cautery used by Keith. Drainage removes the pabulum of infection. In case of very extensive adhesions and positive hemorrhage, the value of the tube cannot be questioned.

The danger involved in inserting a simple glass tube in the abdomen is very small indeed. The involvement of the peritoneum in the perforations of the Bantock tube has been urged as an objection to its frequent use. This danger may be entirely obviated by carefully packing the tube lightly with absorbent cotton, which also, by capillarity, facilitates drainage. The tube should be rotated occasionally and slightly elevated. When kept clean, by frequently changing the cotton, with careful attention to that part of the incision through which it is introduced, there is no appreciable danger of infection. I have reached this conclusion after using it in the most extreme cases of pelvic adhesions, extra-uterine pregnancy, hysterectomy, pus tubes, and so-called simple operations. I have never had a single fatal result which could be attributed to the use of the drainage-tube, and in numerous cases I have seen serious trouble relieved by its introduction.

Irrigation is an important adjunct to drainage in abdominal work. It is of the utmost value in removing dèbris, clots, and shreds. Pouring water through the incision is not so efficacious as its introduction by a syringe, which makes an uninterrupted current. I place the nozzle of the syringe through the lower angle of the incision, and with two fingers retract the intestines, thus giving free exit to the current. In this way, the entire abdominal cavity can be thoroughly washed out.

I cannot see any advantage in Prof. Martin's method of drainage through the vagina. To close the ventral incision to make one in the vagina seems to me unnecessary and illogical. If drainage be decided upon, the site of the incision offers every requisite.

In this connection, I will say that all chemical solutions are unnecessary and positively harmful. Such agents introduced into the peritoneum have done much mischief. The intestinal pain so often following operations in which solutions were applied is, in many cases, due to the adhesions formed in consequence of their use.

In deciding the important question, When shall the tube be removed? we should look to the character and quantity of the discharge. When this is clear and sweet and scant, the tube should be removed. It has been urged that the tube delays union and thereby increases the danger of ventral hernia. This I do not believe. A very long incision, getting up too soon, and discarding the bandage too early, are the most potent causes of this accident. Indeed, I believe the objections urged against the use of the drainage-tube are theoretical and deserve no consideration, in view of the important and serious perils eliminated by its use, viz., protection of the peritoneum from infection. The faith I have placed in it has been fully justified by the results attained.

### A CONTRIBUTION TO THE STUDY OF PELVIC ABSCESS.

By CLINTON CUSHING, M. D., of San Francisco.—He defined a pelvic abscess to be any collection of pus in the cavity of the pelvis which exists in the tissues outside of the cavity of the hollow organs, such as the bladder, uterus, Fallopian tubes, and the rectum. He was becoming more and more convinced each year of the truth of the statement made by Noeggerath, that uncured gonorrhea in the male is a very frequent cause of salpingitis. A recent case came under his observation, in which this theory was verified in every particular. A woman, 27 years old, had been seduced nine years before; since that time she had apparently led a moral life, but had suffered from chronic pelvic abscess, resulting in repeated attacks of pelvic inflammation. She had lately married, immediately following which all her symptoms became very much aggravated; while the husband himself contracted a severe urethritis, which resulted in perineal abscess. Her case becoming serious, he saw her and diagnosticated a probable pelvic abscess. Upon opening the abdomen, the right Fallopian tube was found distended with pus at its larger extremity, and a pelvic abscess containing several ounces of pus. He removed both tubes and ovaries. A microscopic examination showed micrococci present in both tubes, mingled with the pus. The secretion of the urethra of the husband also contained gonococci. This case would seem to demonstrate that the woman had been infected with the gonorrhea nine years before; that its specific character remained in the Fallopian tubes, causing frequent attacks of pelvic inflammation; that the husband had contracted the disease of the woman; that the sexual relations had aggravated the already existing disease of the woman, resulting in the formation of the pelvic abscess, which necessitated operation. She recovered without a bad symptom.

In another case, he opened the abdomen and found that a portion of the abdominal cavity below the umbilicus was an immense sac of pus, containing at least a gallon. A counter-opening through Douglas' pouch was made, and a drainage-tube drawn through the abdominal opening into the vagina. On the second day, the bladder sloughed, the urine escaping into the pus cavity. An artificial vesico-vaginal fistula was formed, that the urine might drain from below, and thus enable the pus cavity to heal. At the end of the year the cavity was healed, the abdominal wound was closed, the vesico-vaginal fistula was repaired, and the patient restored to health.

He presented a dilating trocar which he used in operating in cases where it was necessary to puncture through the vagina. He also presented a self-retaining drainage tube of T shape, made of a piece of rubber tubing of suitable size, the cross-section fastened in place with strand of silk, introduced by pressing the end of the cross-section within the grasp of a pair of forceps, carrying it into the pus cavity and liberating the T. The dilating trocar had proven in his hands a most satisfactory instrument, enabling him to afford speedy relief in many cases, where, without it, procrastination would have been necessary, and much valuable time lost.

Dr. W. W. Potter (Buffalo).—I wish to introduce to the notice of the Fellows of this Association what I think is, possibly, a drainage tube of a little greater advantage than the one Dr. Cushing has presented. Its collar is continuous, is a part of the tube itself, and will keep the lumen of the tube open.

Dr. Joseph Eastman (Indianapolis).—I have one remark to make with reference to the drainage tube and dilating trocar. Prof. Martin, of Berlin, has been using the ordinary dressing forceps, forcing it through the vault of the vagina, seizing the drainage tube, and drawing it up in that manner. I have in that way been using the Bozeman dressing forceps, simply sharpening their points.

I was much interested in the paper of Dr. Price. I believe, in a publication of mine, I made the statement that I would drain upon the slightest excuse; that I still hold to. Now as to the question of permanent drainage. Dr. Wylie, of New York, speaks of establishing permanent drainage. I do not know how that is to be done. In my experience, and that of others, I find that, in at least a week, nature has covered in the tube with plastic material. In the first ovariotomy by Dr. McDowell, he allowed the ligatures to hang out of the abdominal wounds. I am not sure but that is about as good drainage as we ever had or will have. It is good capillary drainage. Since that time, in fifty-one cases, I have not had a hernia except in one case. That was where I had to leave the drainage tube in over a week. It seems to me the entire key to the situation is in bringing the abdominal layers into exact apposition. I am satisfied that we must not decide for vaginal drainage or abdominal drainage, one against the other.

Dr. E. E. Montgomery (Philadelphia).—The members of the association are greatly indebted to Drs. Price and Cushing for their practical papers on a subject of the greatest importance to those practising abdominal surgery. I take it, in the practice of this department, we are desirous of using no measures unnecessarily, and on the other hand of leaving undone nothing that is important for the relief and salvation of our patients. With this in view, it seems to me it is exceedingly important that practical indications should be laid down for the treatment of such cases, to make sure the class of cases in which drainage is indicated and necessary, and outline those in which it is not indicated. In many cases the necessity of drainage may be obviated by the subsequent care of the patient. If we place the patient on a restricted diet, or diminish the amount of liquid, so that the pressure in the vessels will be decreased, and the vessels, instead of throwing out liquid, will take it up, there will be less danger of peritoneal effusion. In the applications of the dressings, where we have reason to expect peritoneal effusion, if we apply pressure, we will find the danger of effusion is lessened. Another important thing is the care of the drainage tube, so that the secretions are not allowed to decompose in the peritoneal cavity. By the application around the drainage tube of the rubber dam, a piece of rubber such as is used by the dentists, making a complete covering over the dressings, we can wash out the cavity without soiling the dressings at all. The drainage tube is objectionable where it is unnecessary, for the reason that it lessens the strength of the union in the wound. It is also for the reason that it increases the difficulty of keeping the cavity pure and sweet. There are cases, I think, in which vaginal drainage is preferable to the drainage from above.

Dr. H. O. Marcy (Boston).—There are two or three points in which I shall draw exceptions in the matter of detail to the gentleman who read the paper. We certainly all understand that we want to get rid of deleterious, effete, poisonous material. The first question the operator places before himself is, that he does it by operative measures. If he is sure he does it, it is a gratuity to add the danger of the introduction of a tube which itself may carry in infection. If it is removed, I am sure he would agree with me that we have nothing to drain, because we have a healthy non-infective cavity. The question comes, Can we do this? If we are in doubt, can drainage accomplish that which you desire? Unfortunately, we all have to answer that many times it signally fails when we have trusted to it. If we had an infectious tube, we would destroy the point of infection before we closed the wound. There is nothing better than to be certain that you follow back the infection to its very center. If you have a doubt about the aseptic properties of the cavity, wash it freely with aseptic water. If you have not removed this, the next question you ask is, Does the drainage tube make your patient safer? The drainage tube often fails. When the drainage tube fails, then the only safety to your patient is a reopening of the cavity. I believe that, if we have to use drainage, we should drain in the direction of gravity and not against it; that is what drainage means if we understand it. I am strongly inclined to think favorably of vaginal drainage. I have designed a drainage tube which I will be glad to present this afternoon, two tubes lying in conjunction, with a diaphragm; then you wash in either way, which I believe is of advantage.

Dr. Albert Vander Veer (Albany).—I scarcely think there will be presented to us any subject of so much importance as this of drainage in the pelvic cavity. It is with pleasure I refer to one man in this country, one working in the direction of drainage in pelvic abscess. Had he lived he would undoubtedly have carried the subject much further along and to a greater degree of perfection than has been done since his death. I refer to Dr. E. R. Peaslee. At one time his percentage of recoveries was greater than any other man's. He excelled Sir Spencer Wells in his percentage of recoveries. His theory was to drain in the direction that is natural for the fluid to flow. In introducing the drainage tube at the lower end of the abdominal wound, I believe it is well to put in your suture in such a way that it can be tightened as soon as the tube is removed. I can only call to mind two cases in seventy operations where I had hernia occurring. One was because the patient got up too soon. In closing up the wound, bring peritoneum in connection with peritoneum, fascia with fascia, skin with skin. They must be brought together carefully. We must select according to the cases the form of drainage to make use of.

Dr. Joseph Price.—In conclusion, I meant simply to discuss actual disease. I was not dealing with simple cases. I have studied most carefully the works of Dr. Martin, Tait, and others. When I consider the mortality of twelve in seventy-two for pelvic surgery, I must say that I do think Dr. Martin needs a little further experience in pelvic drainage. If you refer to Bantock and compare his mortality record, I think you will consider that it is wise to practice the drainage of Bantock, of Tait, and of Keith, and not that of Martin.

In regard to hernias, you can pick them out from a class of operators that make long incisions. An incision that is long enough to admit two fingers is quite sufficient. You can do anything in the pelvis with two fingers that you can do with the whole hand. The capillary drain I do not look upon as of very great importance. The frequency of cleaning the tube is of paramount importance. You cannot get along without a nurse with special training. A word with regard to the aspirator. I used it a few years ago; I have no need for it now. It is a very easy matter to keep a tube clean, sweet, and dry. I would call attention to eighty-four cases done in the alleys and courts, without carbolic acid or bichloride of mercury, with one death in eighty-four. I simply challenge the world to beat it. The nurses were all trained to clean those tubes. I have had no experience with vaginal drainage; the other has served me well. The tube is a little sentinel to show when hemorrhage occurs.

### AFTERNOON SESSION.

Dr. William H. Meyers, of Fort Wayne, Ind., presented a paper on the

### TREATMENT OF SUPPURATIVE PERITONITIS, in which he asserted that he knew of no reason why the same general principles should not apply to the largest serous sac in the body, where life is in danger from the irritant nature of its inflammatory contents, as holds good when pus has formed in the brain, in the pleural cavities, joints, or cellular tissue; namely, that all these conditions are amenable to surgical interference at the earliest possible moment when we have arrived at an exact diagnosis. He distinctly emphasized that he did not believe that peritonitis was ever idiopathic or spontaneous in its origin; nor that the peritoneum was ever the seat of independent inflammation, but that it was peculiarly liable to inflammation of a secondary character, the irritant proceeding from without, as in wounds, or from one of the organs lying underneath the membrane; or from injuries to the abdominal parieties, rupture of hydatid, ovarian, or other cysts, the bursting of an abscess, perforation from the diseases of the hollow viscera; these being the most common causes. Regarding ovariotomy as the parent of peritoneal surgery, he presented a résumé of a few cases in support of the idea advanced by Mr. Barker, where ovariotomy was performed during the progress of acute peritonitis, and quoted Mr. Wiltshire as saying that he was able to show, many years ago, that the presence of peritonitis is not always a bar to operations. On the contrary, it may be the chief indication of the necessity for operation, being itself due to acute cystic changes, and subsiding only on their removal. Accepting this principle, he believed that attempts at radical cure might justifiably be made, in proper cases, even in the presence of peritonitis. Referring to treatment by the trocar and aspirator, he quoted the following letter from Professor Thomas Anandale, which is believed to be an account of the first authenticated case where the trocar and washing out of the abdominal cavity was resorted to as a curative measure in suppurative peritonitis: "Edinburgh, August 16, 1888. Dear Dr. Myers—In the year 1860, when House Surgeon in the Old Royal Infirmary of Edinburgh, I was asked by the late Prof. Hughes Bennett to treat a case of suppurative peritonitis in a man aged about fifty. Prof. Bennett desired me to place the man in a hot bath, to tap the abdomen under water, to drain off all the pus, and then to pump hot water into the peritoneal cavity until the water came out clear. I carried out the proceeding exactly as he wished me. The patient died several hours after. But this is certainly one of the first, if not the first, cases of surgical treatment in this disease, and Professor Bennett appears, therefore, to have anticipated what is now a recognized procedure."

After summarizing four other cases, treated after a similar method, he proceeded to consider the treatment of purulent peritonitis by incision. Treeves says, True has collected ninety-seven cases of deliberate surgical interference with the peritoneum, and this numerical statement alone suffices to show the rapidity with which the treatment has spread. To the above list he would add the following reported cases: Two by Thoroughgood in 1885, one by Clark in 1887, two by Oberst, one by Studensky, one by Robertson, one by Halliday Croom, and finally to this list he added one of his own cases, in which he made laparatomy for wandering spleen lying in a peritoneal abscess. The spleen was removed, drainage cavity washed out with carbolized water daily, recovery. To this list might be added ten cases of laparatomy for chronic and acute peritonitis reported by Lawson Tait in his work on Pathology and Treatment of Diseases of the Ovaries, 1873. He asserted that by far the most frequent cause of suppurative peritonitis was perforation of the vermiform appendix. Where it occurs, the only chance of life lies in opening the abdominal cavity early, and thoroughly washing out and draining. This is the treatment whether the peritonitis is general or local. Considering the method of operating, he thought no definite time could be fixed. The urgency of the symptoms alone were to considered. Should the median incision be made or should it be made immediately over the cecum? Marcus Beck believed the incision should be over the outer part of Poupart's ligament, and should be three inches in length, which would enable the surgeon to reach the point more readily, particularly if deep-seated and low down in the cavity. After thoroughly cleansing the abdominal cavity and inserting a large size drainage tube, the treatment is complete. He closed his paper by two quotations, the first from Lawson Tait, in his address before the Gynecological Society of Great Britain, upon the question of operative treatment in puerperal as in other forms of peritonitis: "I have now come to the deliberate conclusion that it is an act of almost criminal omission to allow a case of peritonitis to die without the abdominal section." Again, in a letter of recent date from Mr. Greig Smith, of Bristol: "That in cases of suppurative peritonitis, the peritoneum should be treated for a few days in a hot aseptic solution; by keeping the intestines bathed in an innocuous or mildly antiseptic fluid, peritoneal adhesions are prevented."

It might seem to us that we had arrived at ultimate truth, but let us not forget that the fact of to-day may be revolutionized to-morrow by some new appliances for research or some fresh experiment.

Dr. E. E. Montgomery, of Philadelphia, read a paper on

LAPARATOMY IN PERITONITIS, in which he said: The dread of disturbing the peritoneal cavity has been so great as to well justify the assertion of Wagner, that he and his contemporaries had been nurtured in the fear of the Lord and the peritoneum. Peritonitis varies in its phenomena according to the condition producing it. It is recognized that the disease, in the idiopathic form, can arise from exposure to cold and dampness, in which the exudation is limited and likely to be serous. In other forms, exudation is more likely to become purulent, or there is an early secretion of pus. The pus may be free, or become encysted.

The largest collections are more likely to occur in the acute disease, and especially in the puerperal and metastatic forms. According to Bamberger, the exudation, in its beginning, is fibrinous, but it is evident that there is a primary pus formation, as the cases quoted by Abercombie and Kaiser show where pus was found after thirty-six to forty-eight hours of inflammation. The removal of pus is as imperatively indicated as when it occurs in pleural or articular cavities.

The presence of pus is indicated by fluctuation, dull percussion, attacks of chills, hectic, cessation of spontaneous pain, sentiveness upon contact and movement. The diagnosis may be confirmed by puncture with a hypodermic or small aspirator needle. Pain is not always present in peritonitis, though pus may be present in large quantities. Non-purulent effusion may be removed by puncture, but suppuration positively indicates laparatomy, as it allows of thorough evacuation of the pus, complete cleansing of the abdominal cavity, separation of adhesions, the removal of exudation, and perfect drainage. It also permits of thorough examination, and may enable us to discover and remove the cause of the inflammation.

In perforative peritonitis, the operation should be done in all cases in which collapse has not already taken place, though the result will be more favorable in the cases of traumatic origin. In these, the patient is usually healthy, tissues of the viscus are in good condition, and sutures are readily borne, should resection be required.

The class of cases demanding early attention are those occurring about the region of the cecum. These cases arise generally from perforation of the appendix. Fitz says that thirty-four per cent. die within three days. In severe cases, laparatomy should be performed at once; and when gangrenous or perforated, the appendix should be amputated and the stump sutured. The lateral incision is preferable to the median.

In no form of peritonitis has laparatomy proven more valuable than in the tubercular. Its value was discovered by accident, the operation being done under mistaken diagnosis. The favorable result is attributed to the removal of the serum which serves as culture-fluid for the multiplication of the bacilli.

Incision, in these cases, is preferable to puncture, as ascites does not recur after the former, while it rapidly accumulates after the latter.

In conclusion, peritonitis should be regarded as a positive indication rather than a contra-indication to operation.

Death, in the cases of which we have spoken, is certain; we have seen that the operation may save the patient. Why hesitate to give the latter some chance of recovery?

Dr. Henry O. Marcy.—I consider it a privilege to rise and express my thanks to Dr. Montgomery. It is now nearly two years ago since I opened the abdomen in one case for exploration, because of a considerable thickened mass post uterine. The patient had been six months under observation and gradually became worse. The diagnosis had not by any means been certain. I do not suppose it had occurred to any of us that she had tuberculosis, but the exploration showed that the whole peritoneum was studded with those little masses which are so familiar to you all. There was in this instance no especial amount of serum. The abdominal cavity was somewhat carefully washed out with a sublimate solution. Before closing the cavity, I cut off a little portion and submitted it to Dr. Nelson, who made not only a careful microscopic study, but cultivated the bacilli by artificial process, so that there was no doubt about the condition. But, strange to me and to you, the patient from that moment began to improve and is now comparatively well.

Dr. Clarke.—Dr. Marcy invited me to see a case in which exploratory incision was done very late. Had it been done earlier, I think it would have been successful. I have had an opportunity of seeing a good many such cases since, and I hope and have encouragement to believe that it is a feasible operation. Not long since, I saw a young girl who had evidence of phthisis and bacilli of the intestine, who, after a very small exploratory incision and draining off all the serum, seemed to get well in a short time. There was considerable serum in the case.

Dr. Ricketts (Cincinnati).—The discussion of the treatment of peritonitis by abdominal section has not taken the stand here that I hoped for. It is a very important subject, and I feel that the death-rate of peritonitis is going to be greatly lessened in the next few years by abdominal section. The trouble in these cases is to have the general practitioner understand the gravity of these cases early enough. It is not always the abdominal surgeon who sees the case in the beginning. Many times a bad result is counted against the surgeon, not for any failure on his part, but by failure of the case being recognized at the proper time. I feel that it is the duty of every abdominal surgeon to do what he can in order to get the general practitioner to understand when to call assistance in these cases. I think it is our duty to give them to understand in every way possible the importance of early interference in these cases.

Dr. Thomas Opie (Baltimore).—A remark has brought to my remembrance a case, a single one in my experience, in which I was called in by two physicians; I think on the fourth day after labor. The patient was then in a very bad condition, in a very critical state. I asked immediately if there was not some traumatic origin of the difficulty. They insisted that, so far as they were able to judge, there was none. I insisted on a digital examination and found there was a rent in the right side of the cervix, in which, by some very remarkable accident—possibly one of the blades of the forceps was not introduced into the os—the uterus was torn very severely. Four fingers of my hand would go into a deep rent. I said to the gentlemen that I thought she was almost moribund, but that it was worth while to try something; it was a certainty that death would ensue, but something was left and that was laparatomy. Appreciating the gravity of the situation, it was insisted by the friends that we should give this one chance. I opened the abdominal cavity and found a large quantity of serum and many adhesions. The patient died ten or twelve hours after the operation. I mention the case because it was one in which I think it was right to give her one chance out of a hundred.

### THE RELATION OF THE ABDOMINAL SURGEON TO THE OBSTETRICIAN AND GYNECOLOGIST.

By Dr. A. Vander Veer, Albany, N. Y.—He said that in studying the constitution, by-laws, and make-up of this society, he observed that there were brought together elements which, if properly harmonized and "the principle of unity and research" preserved, could not fail to benefit ourselves as individual members; and also that the influence which will go out will be for good to the profession throughout this broad land. He would endeavor to bring out the points of the specialty which he represented in such a manner as might result in mutual good to all. He had been led to ask the question, "Is there such a department as abdominal surgery in our profession?" That was a grand union when surgery, freed from its barbarism and ignorances, and no longer associating with or being part of the "Barber's company," united in honorable wedlock with medicine, and helped to establish that household in the healing art from which many of us who are here to-day have seen springing and going out the most of the grand specialties that have done so much for suffering humanity. Prof. Virchow said: "Within twenty-five years the great host of specialties has developed; and it would be vain, anyhow fruitless, to oppose this tendency; but I think I ought to mention it here, and hope that I shall be certain of approval when I say that no specialty can flourish which separates itself entirely from the common course of science; if it do not take the other specialties into account, and if all the specialties do not mutually assist one another." All of this positive and signal advance has been made, not in a spirit of hostility to general medicine, but to render aid to one another, as we work against disease. Surgery has not accomplished it entirely alone; she has had the assistance of true medicine, but in doing what she has done, she has undoubtedly advanced medicine in a very great degree.

Of those who remember the crusade which was early made against ovariotomy, which was but the beginning of abdominal surgery, and who cherish the belief that the latter is not yet a distinct part of general surgery, the question is asked, "Why is it that special works are being published on abdominal surgery alone, and meeting with ready acceptance by the profession?" Abdominal surgery may be said now to be a part of the specialties of obstetrics and gynecology. Has not the work of such men as the late Prof. S. D. Gross, Tait, Parkes, Sands, Weir, Senn, Greig Smith, Bull, and a host of others now living, demonstrated the fact that abdominal surgery has become a specialty? If we inquire into the manner of its development, we find that it has been the outgrowth of work done by the general practitioner. The elder Gross once stated that his experience and observation had taught him that they made the best specialists who, having engaged for a number of years in general practice, finally confined themselves to that special department for which their studies, inclinations, and clinical observations and experiences had best fitted them. These words apply with peculiar reference and force to him who attempts to practice as an abdominal surgeon. The operation for removal of a simple uncomplicated ovarian cyst is probably the easiest operation that occurs in all the range of the major operations in surgery, but when we come to the operation, embarrassed as it may be by all the complications now known to us, it becomes one of the most difficult of all operations. Notwithstanding all that has been done within a short time by obstetricians, gynecologists, or abdominal surgeons, to advance abdominal surgery, we are yet at the half-way house on the road leading to complete success, for not yet is made clear the best manner of operating, the best place for the operation, or the best way of dealing with all the various complications. Our entire profession must yet be taught much that pertains to abdominal surgery, and it must to a great extent be done by men of creative genius. What we need is not the elaborate presentation of theories, but the offering of practical papers that will give facts, and the accumulated wisdom and experience of honest workers.

Specialism in society work and in journalism should be encouraged by the profession at large. As abdominal surgeons we should move carefully, but at the present time we must be active and on the alert to make use of the constantly increasing material that presents, and from which to draw our practical deductions. In this new society we have formed a combination that illustrates well that trite but true saying, that in union there is strength. The work of the obstetrician is the most arduous, important, and responsible of any part of our profession. The obstetrician and abdominal surgeon should work in perfect harmony. The latter bears to the former much the same relation that the general surgeon does to the neurologist in his brain surgery. The obstetrician, like the neurologist, will make his diagnosis, but the surgeon will often be called upon to do the operation. The work of the abdominal surgeon will necessarily lead him into the field of gynecological practice; that is, the major portion, not the minor part, of gynecology. There are many good gynecologists who are successful in everything that pertains to their specialty aside from doing an abdominal section, and this they assign to some one else. I believe it requires years of the hardest study and effort to become a thorough abdominal surgeon; the number must ever be somewhat limited.

Finally, is it not a fact that there is some danger that abdominal section may be carried too far if made too much of a specialty? Have we not in this society the conservative combination? That our new society is moving in the right direction, witness the new obstetrical and gynecological society that has just been organized in Vienna, and of which Prof. Breisky has been elected president.

Dr. S. C. Gordon, of Portland (by invitation).—I hardly know how we can discuss a paper like this except to assent to what has been said. The paper has certainly covered the ground. A discussion, in order to be interesting, must take a departure from the subject discussed. Abdominal surgery in my opinion is now taking quite a different turn from what it formerly did. Formerly we believed that everything of this nature must be done in a special hospital, with special accommodations. My own experience justifies me in saying this that, while we must take every possible precaution, we must not allow a patient to die because we cannot remove them to a special hospital. The question of special hospitals is no longer a sine qua non.

### THE SURGICAL TREATMENT FOR LACERATIONS OF THE PERINEUM AND THE PELVIC FLOOR.

By Dr. W. H. Wathen, of Louisville.—He spoke especially ot the surgical treatment of lacerations or injuries of the muscular and aponeurotic structures that form the floor or diaphragm of the pelvis. He said there is probably no other subject in gynecology about which so much has been written that is of no real value, and that a relatively simple operation had been made to appear so complicated that it is seldom correctly performed. He passed by much of the immensity of pseudo-scientific rubbish, and took a practical view of the subject.

He said that the muscles and the fascia in the perineum give it strength, and, when they are lacerated, no operation that does not primarily tend to reunite the torn ends is logical, or will be followed by permanently good results. We may have prolapsus of the uterus, with rectocele and cystocele, resulting from subcutaneous rupture of these structures, with no laceration or injury of the mucous membrane or other parts of the perineum. This condition is usually not diagnosticated by the attending physicians, and the woman is subjected to various plans of treatment to hold the parts in position and relieve the annoyance from pressure, weight, etc., all of which give but little relief; nor can we cure her except by an operation to bring together and reunite the torn ends of the muscles and fascia.

He said that when any or all of the perineal union of the muscles, or of the fascia, is lacerated, unless at once united and held together, the muscular contractions continue to widen the distance between the torn ends, so that the vulva gradually becomes enlarged laterally. The extent of this lateral separation is governed by the degree of laceration and the length of time since it occurred. If the above is correct, then no operation will succeed that fails to bring these torn ends together so as to reunite them. This is a simple question that holds good in all operations to restore the perineum in complete or incomplete ruptures, and if we are controlled by it, and are familiar with the technique of the operation, success will nearly always crown our efforts.

He did not know of any operation that is not faulty in this particular, but the operations that accomplish this purpose best are performed by Tait, Duncan, Simpson, Langenbeck, Saenger, Hart and Barbour; but if he understood their methods correctly, they do not fully appreciate the importance of dissecting up and uniting the muscles and fascia. This cannot be done by the usual method of denudation, but is accomplished by a splitting process. The incisions should go deep near the anus on the lateral borders of the vulva, and the recto-vaginal septum should be split through the connective tissue between the vaginal and rectal layers, so that the vaginal flap may be thick enough to prevent sloughing.

He did not think it necessary to give the reasons why the primary operation should be performed, as there are now but few men of recognized ability in obstetrics or gynecology who are opposed to it, and we are not a little surprised to find in this list the name of the distinguished professor, A. Charpentier, of Paris. His objections are illogical, and are not sustained in actual practice when the operation is correctly done. He had done the primary operation often himself without a failure; in fact, he thought the success is usually more perfect than in the secondary operation. The torn ends of the muscles and fascia are now easily held in apposition, and unite within a few days. He reported a typical case in which he had operated a few weeks ago for his friend, Dr. ——. The woman was delivered of a large child when sixteen years old, was torn through into the rectum for over an inch, and the vaginal wall and the connective tissue were torn two inches farther up. The operation was done about one and one-half hours after delivery. He used fifteen sutures in the vagina and the perineum. The vaginal tear was united by silk sutures, and the perineal by silver wire and silkwormgut, using only one silver wire as a base suture to hold together the ends of the sphincter muscle. The sanitary and hygienic surroundings were not good, and she had but little after-attention. She passed her urine normally, the vagina was washed out but a few times, and her bowels moved daily after the second day. At no time was there any pus, and the entire laceration healed by first intention.

If the operation is well done, he doubts the necessity of drawing the water or tying the legs. Nor is it necessary to wash out the vagina often. The urine and the lochia are not poisonous, especially after the second day, if strict asepsis has been observed in the operation. Where any form of an aseptic animal suture is used, the needle should be introduced and brought out just within the lower or external edges of the raw surfaces, so that, when they are united, the sutures will be concealed or buried in the tissue. Sometimes a few superficial sutures will be required. The suture should be so introduced as to be entirely covered by the tissues, and to bring the surface into even and exact apposition. If the sphincter ani is ruptured, he always uses the base suture, after the fashion of Emmet.

He does not destroy any tissue except jagged edges in some complete ruptures; the dissected part assists in protecting the wounded surface against the dangers of infection from the uterine or vaginal secretions, and also increases the thickness of the perineum. He had never had a recto-vaginal fistula after an operation for complete rupture, nor did he believe it will often occur, if the operation is correctly done, after his method.

Dr. Henry O. Marcy, of Boston, read a paper upon

### THE PERINEUM: ITS ANATOMY, PHYSIOLOGY, AND RESTORATION AFTER INJURY.

He reviewed carefully the teachings of the text-books and the views held by various authors whose writings are found scattered through the medical journals. These he supplemented by a series of photographs of dissections of his own work, from which he verified, in large measure, the teachings of Savage. He especially emphasized the importance of a better general knowledge of the anatomy of the pelvic structures, and pointed out where the teachings of many of the text-books are in error in important particulars.

Dr. Marcy considers the floor of the pelvis, in its normal relations, a greatly strengthened by the transversus perinei, which not only aids the levator ani in all its functions, of which, indeed, it may be considered a part, but that it holds in a fixed relation the pubo-coccygeus —the muscular loop which extends from the pubes and grasps, so to speak, the anal opening, crowding it forward upon the vulvar outlet and this in turn upon the urethra. This preserves the anterior forward curve of the vagina, and upon it depends the intra-folding of the vaginal muscle, making the letter H in section.

Dr. Marcy claims that this peculiar disposition of the lateral intra-folding of the vaginal canal is the chief factor which gives to it the elastic support, quite sufficient to retain the cervix in normal position. The transversi perinei once sundered and, by their retraction towards their origin, the plane of the constrictor group is so elevated that the vulvar opening is drawn backward, eversion of the posterior wall now ensues, with its more or less complicated train of nervous and pathological complications. The injuries of the fasciæ were also carefully considered, but relegated to a position of importance quite secondary to that of the muscular groupings.

For the better understanding of the various methods recommended for the restoration of the pelvic floor, Dr. Marcy projected upon the screen a large series of illustrations and analyzed them, thus pointing out the modifying influences which have led up to the later modes of operation. Although the deep muscular floor of the pelvis is the avowed object in restoration, the methods, almost without exception, are confined to a dissection of the mucous membrane covering the original muscle and a refreshing of the vulvar cicatrix. This, for the cure of rectocele, produces a folding in of the vaginal muscle in varying pattern, and by deep stitches, taken almost at random, there is brought together in the median line the connective-tissue raphé. While it is not denied that this may sometimes give good results, as a rule the cure is not complete and is faulty, in that the reconstruction is defective.

The object clearly should be a restoration to former condition, and this means a reunion of the sundered muscles and fasciæ, instead of puckering the deformed sheath of the vagina, at most an easily distensible canal. To accomplish this, Dr. Marcy devised his operation, which surely is to be commended for simplicity and effectiveness. With two fingers in the rectum to put the parts on tension, he detaches the vagina in its posterior third from the vulvar outlet and carries up the separation in the recto-vaginal space as far as the upper border of the rectocele and laterally into the sulci. The flap thus made is lifted by an assistant, then the surgeon carries a large curved needle deeply into the posterior fasciæ laterally and in a continuous stitch (quite unlike the over and-over stitch), with animal suture, he closes, from side to side, posterior to the vagina, the stretched fasciæ. Having in this way remedied the rectocele, the sundered, separated ends of the muscular group are approximated by a continuation of the deep-buried suture. The external wound is coapted by a fine continuous suture taken as a "blind stitch," which is also a buried suture, and the surface is covered with iodoform collodion.

Dr. Marcy prefers to use the deep continued stitch by means of threading the suture through the eye near the point. The needle is then introduced thus threaded, is carried through, the suture unthreaded, the opposite end of the suture threaded, and the needle withdrawn, carrying with it the suture. This makes an *even, double*, continuous stitch and secures a much better closure of the separated parts. If the parts seem tense, which is rarely the case, a double pin is placed outside the sutured structures (formerly described by Dr. M.) to hold the parts, without strain, at rest.

When the laceration is complete, Dr. Marcy divides the rent laterally and, in the manner already described, coapts with a fine, buried, continuous animal suture the rectal and vaginal sides, and then proceeds to bury his deep sutures as in incomplete lacerations. He showed, by quotations from his previous publications upon the subject, that he was the first to practice and recommend the use of the deep-buried animal suture in perineorrhaphy, published by him in 1883, although this practice has been attributed to Schroeder, who first published his method in 1885. Dr. Marcy uses tendon from the kangaroo as quite superior to catgut; this is prepared so as to be entirely trustworthy, but emphasis is made upon special caution to render the operation aseptic, in this portion of the body especially difficult, and without which the operation will be quite likely to fail of cure. He always practises the greatest precaution and has had most excellent results.

---

### SECOND DAY—MORNING SESSION.

### DOUBLE OVARIOTOMY DURING PREGNANCY.

By Dr. W. W. Potter, of Buffalo. [This paper appears in full in the *American Journal of Obstetrics* for October, 1888.]

Dr. Vander Veer.—Mr. President, Dr. Potter has presented just that kind and class of cases we ought to have given us. This is an exceedingly rare case. I have looked up the subject somewhat hastily, and find ninety-six cases reported where the operation has been done during pregnancy—not *double* ovariotomy. It is an operation perfectly justifiable. The condition of pregnancy does not seem to interfere very much with abdominal operations. The case he referred to I operated on about three months ago. I had a letter from the physician in charge saying that she is progressing nicely, but I do not care to have my case go on record yet.

Dr. E. E. Montgomery.—I was very much interested in this case of Dr. Potter's. Two years ago I read a paper on Ovariotomy during Pregnancy. I was able to find a record of sixty cases, and showed that pregnancy had very little influence upon the mortality of the operation. In all those, there was not one in which the operation was done upon both ovaries. In the report of Dr. Munde, I think, it was claimed that a woman was not likely to go through gestation with both ovaries removed. This paper of Dr. Potter shows that this belief is erroneous. I had thought, until Dr. Potter made this report, that my case was the first. I performed an operation last November, removing both ovaries at about the third month of pregnancy. The patient was confined at full term, the child weighing eight pounds. There were no unfavorable symptoms whatever. The theory of Tyler Smith was that the ovaries directed the contractions of the uterus in labor. These two cases having gone through the full term, and having, at the completion of gestation, the labor entered upon and conducted in its normal manner, indicates that this theory is not perfectly correct. We must look for some other cause for the induction of uterine contractions at this time.

DR. MAXWELL, of Keokuk.—There is one other point that is interesting; that is, that the patient operated upon nursed her child after gestation had been completed—another fact, that lactation is not a substitute for menstruation.

DR. BYRON STANTON, of Cincinnati, read a paper on the subject of

INDUCED PREMATURE LABOR.

He referred to the arguments which have been urged against this procedure, based upon the ground of its morality and usefulness—arguments that would apply with equal force against all obstetric operations. He thought that, like the obstetric forceps and version, it would tend much to do away with craniotomy and Cesarean section. He thought that the arguments that the mother is exposed to some risks that she does not encounter at term, such as imperfect development of the uterus, incompleteness of the changes which nature effects in the maternal passages during pregnancy, the frequently delayed detachment of the placenta, more frequent malpresentations, etc., were more than met by the advantages gained by the smaller size of the child, and more yielding and compressible head, and consequently shorter and safer delivery. He also referred to the arguments against induction of labor, on account of the great infant mortality, quoting some recent writers as holding that such intervention is so dangerous that the operation should give place to Cesarean section—an operation that has rapidly reduced its mortality. He thought the conclusion not justified by statistics.

The points involved in determining the question as to the resort to the operation are:

1st. Whether the case is one in which delivery at term will involve the death of the infant, and subject the mother to much dangers and suffering, and yet permit delivery at seven or eight months.

2d. Whether the conditions are such that the life of the mother will be imperiled by continuance of pregnancy to full period of gestation, by hemorrhage, or some intercurrent disease, or by aggravation of some chronic ailment which can be averted or removed by earlier delivery; and

3d. Whether there is a history of infant fatality in several successive pregnancies at some regular time after the age of viability.

Dr. S. considered the different conditions which might call for interference, as contracted or deformed pelvis, unavoidable hemorrhage, eclampsia, persistent vomiting of pregnancy, etc. He referred to the difficulty of fixing extremes of measurement that would limit the operation. Absolute limits cannot be fixed, the propriety of the operation being determined as much by the size of the fetus as the contraction of the pelvis. The difficulties of labor depend, to some extent, upon the character and location of the deformity. Delivery is more easily effected through a flat pelvis than through a generally contracted pelvis, with the conjugate of the same measurement. Deliveries are easier, other things being equal, when the contraction is at the superior strait than at the inferior strait. If both straits are contracted, the difficulties are much increased, for uterine action might overcome resistance at one strait, but be unable to overcome it at both extremities.

There are two difficulties in these cases; one is in determining the period of gestation, but, he thought, counting from the last menstruation, and the period of quickening taken in connection with the size of the uterus, would give a sufficiently accurate estimate in nearly, if not quite, all cases. Another difficulty is the determination of the diameters of the pelvis in living subjects. Pelvimetry is not an exact science, and the instruments devised for measurements of the pelvis do not give exact diameters; he thought a well-practiced finger as good as instruments, and would give sufficiently accurate measurements.

Reference was made in the paper to the differences of opinion among obstetric writers, in regard to the induction of labor in puerperal convulsions, persistent albuminuria, placenta previa, obstinate vomiting of pregnancy, etc. He thought it not necessary to consider at full length all of the conditions which might call for the operation, but gave the general statement that whenever the condition of the mother or child renders the continuance of pregnancy to full term dangerous to both or either of them, and the danger can be removed by delivery in the seventh or eighth month without the substitution of a greater danger, the induction of labor is not only justifiable, but is a duty of the physician. If it is not indicated to save the life of mother or child, it is criminal.

He next considered the methods of inducing labor, of which there are many.

Medicines acting through the general system were condemned. All oxytocics are unsafe for inducing labor, so also are those methods that act by reflex irritation. Some method acting directly on the uterus is to be preferred, and only those are to be thought of that respect the integrity of the membranes. Electricity in its various forms has proved disappointing. The hopes that were at one time entertained have not been borne out by experience.

Kiwisch's, Kluge's, Cohen's, Hamilton's, Lehman's, Schiele's, Krause's, and other methods were described, preference being expressed for Krause's method—the introduction and retention of a flexible bougie between the membranes and uterine walls until uterine action is excited.

In placenta previa he would use a tampon in the vagina, or a Barnes dilator in the cervix, until sufficient dilatation is effected to permit version.

All methods that bring on uterine action by evacuation of the liquor amnii are to be avoided.

After labor, the child demands especial attention. It must be kept continuously warm by artificial warmth. The bath usually given to the new-born *must be omitted.* Early feeding must be resorted to.

Dr. Thomas Opie.—I think the attention of physicians ought to be called to the importance of the principle of taking advantage of that period of intra-uterine life in which the sutures are yet wide and the bones not well developed, and in which we can have this very desirable condition of moulding as the head is passing through the canal. It is remarkable the ingenuity with which nature moulds the descending head to conform to the channel through which it must pass, but this is a process which requires an amount of time proportionate to the difficulties to be overcome. If the contraction is great, the time must be lengthened. If the contraction is too great, then delivery must be impossible if the patient has gone on to full term. I wish to speak of the great importance of taking advantage of the undeveloped condition of the head of the child. We will find the shape of the head indicating the direction of the force, and, so, distinctly pointing out what has been the difficulty and cause of delay. We have to deal with two distinct classes of cases. In one class, after inducing labor, we can wait for Nature to carry it through, but, in cases of eclampsia and placenta previa, delivery must be accomplished at once. Still we should take advantage of the condition of the head, for, with the forceps upon it, we find it still qualified to adapt itself to the canal, but in a better way than at full term. In cases of eclampsia, I think many lives are lost on account of delay. It has not been my experience that the operation of dilating the uterus is either difficult or dangerous. The introduction of hard-rubber dilators will furnish the beginnings. It will soon be found that, after introducing one after the other, the contractions begin, whereas, with the bougie, it might be delayed two or three days. I merely introduce these as a forerunner of Molesworth's or Barnes's dilator, until I can get in two fingers; and then I am able to introduce Taylor's narrow-blade forceps, and with these dilate the os sufficiently to apply the Tarnier forceps.

I would emphasize what the doctor has said with reference to the question of ergot. There is one point about ergot which should be thoroughly established in the minds of the whole profession—that there is great danger to the child from its tonic action, and, as far as the mother is concerned, we start a force that we cannot control.

Dr. A. L. Smith, of Montreal.—I wish to urge the induction of premature labor in bad cases of vomiting in pregnancy. I have a record of eighty cases of death in this comparatively simple condition. I know of one large family of beautiful girls in Montreal, every one of whom was born at seven months.

IS THE FREQUENT USE OF FORCEPS ABUSIVE?

By Dr. Thomas Opie, of Baltimore.—There is a remarkable unanimity of opinion in the teaching of recent text-books, medical journals, and medical societies on this subject.

They favor the more frequent use of forceps, and condemn the so-called expectant management of labor. The axiom "meddlesome midwifery" has beaten a retreat before the clamor for the forceps.

The name of forceps points to its most striking characteristic, its prehensile power. Three distinct eras may be traced in this branch of medicine, having their pivotal influence in the forceps.

1st. The introduction of the original forceps in 1700; 2d. The important invention by Levret of the pelvic curve in 1747; and 3d, when in our own times, 1877, Tarnier invented the traction-rod attachments to the forceps.

Baudelocque arranged methodically the pelvic positions and presentations in 1791. Prior to that time the sovereign powers of the forceps were greatly abused. Their introduction was a matter of haphazard and convenience. Even in the days of Ramsbotham there was a bitter controversy as to which was preferable, forceps or craniotomy.

A recent writer says "the forceps is simple in construction, easy of application, wonderful in power."

The successive stages of their development have, on the contrary, made them far less simple in construction; they have a wider range of application and wonderful powers for life-destroying as well as life-saving.

All surgical operations are dangerous; it is equally true that elements of danger lurk about all cases of artificial labor.

To substitute traction for contraction; to introduce and use instruments in the genital tract; indeed, to substitute art for nature's peculiar and inimitable methods, is always dangerous.

The most unanswerable argument against frequent use of forceps comes from the gynecologists. Good surgeons are relatively few, and delicate and dangerous operations in that branch are relegated to specialists among them, but most practitioners think themselves able to cope with any forceps case. If skill in the use of forceps did not exist prior to Baudelocque's classification, how can we accord this virtue to operators with the forceps in these days, who are ignorant of the fundamental principles and admirable system upon which the present science of obstetrics rests?

The opportunities for the student of obstetrics are meagre; and granting he is thorough in the theory of this department, he forgets it before he is intrusted with a practice. No wonder if he makes a medium-rate obstetrician, and sneers at the mechanism of labor.

To determine between the high forceps operation and version, to diagnosticate or rectify position above or at the brim, to apply the forceps accurately and conduct safely the little passenger through the passage-way, are among the great achievements of medical science.

The high forceps operation is a critical one, and the operator should be chosen with as much care as for an ovariotomy.

The low operation is not so simple as to be left to "any tyro."

For practical purposes, it answers to designate all kinds of forceps in the order of their seniority of invention, as follows: the straight (Chamberlyn), curved (Levret), and traction-rod (Tarnier).

It is high time to turn a deaf ear to the many names of individuals attached to forceps, because of various modifications of lock, blades, or handles. They obscure the horizon of the subject; they distract attention and confuse the object, which, like that of the practice of medicine in all its departments, is life-saving, curative.

There is but little need for straight forceps. The traction-rod forceps alone serve our purposes with satisfaction, at and above the superior strait.

The welding and unification of the traction-rod and curved forceps in Tarnier's latest style well-nigh disarms criticism. It is a great achievement; one on which we may congratulate this age.

Think of the struggle, and pity the sufferings of the man, woman and child as the operator toils away with the straight forceps at the inlet. The pelvic curve added would only qualify, not remedy, the situation.

Still another modification, and science has triumphed. The traction-rods, with relative ease and far less force, pilot the head through the upper narrows, and thence into the safer region of the low operation.

If we could only persuade operators of the salvation of traction-rods covering all the indications for traction at the superior strait; of making traction, during the pains, rythmical, direct, slow; of supplementing, not superseding, nature; of utilizing all the vis-a-tergo and only just enough of his power to bring the combined forces to the normal, and that all above that is abuse.

If we could get them to be careful, to halt, prospect and protect the sphincters of the uterus and vagina, and to beware of rapidly unloading the organ, we would have a reform that would be as life-saving stations all along the passage-way.

I am fully convinced of the ability of the forceps to help. I protest that they should not be used regardless of the factors of delay.

I plead for their judicious use.

As to the indication that the os should always be dilated or dilatable, judgment often goes to protest.

About two years ago, I was in attendance upon a most tedious, nervous, and impatient primipara, in whom the os was only three-quarters dilated; after a boisterous struggle of two nights and two days, slowly, almost imperceptibly, the dilatation increased. The patient's qualifications for abuse, however, grew more rapidly; I came in for a large share of it. She demanded chloroform and instrumental aid; the husband seconded her claim. The traction-rod forceps were applied; she was delivered in two hours under chloroform. A febrile trouble and tardy convalescence ensued; some months afterwards she became the patient of a clever gynecologist. His operation told the tale of the "blundering obstetrician." I have never had an opportunity since to call the patient to an account.

One of the most important requisites for a good obstetrician is "to know how to wait and do nothing." In the first stage, he must wait a long time, especially in primiparæ, before using forceps; indeed, he is not warranted in resorting to it, except the mother or child is in jeopardy.

Far the most frequent use of forceps is in the second stage, and we are told "any tyro" can perform this operation. Nature can generally do it better and safer, if not so quickly. Time and patience cannot be too much extolled as virtues during the whole of labor.

Let the attendant occupy the position of "watchful expectancy." Remove reflex disturbance, sustain by proper food, guard against fatigue, secure rest, assure and cheer, utilize the simpler methods of assistance; let nature do all she can before we resort to forcible measures. I have lately limited the number of my forceps application by manual pressure, according to the plan advised by Kristeller, of Berlin.

This and other manual helps are more in accord with nature's laws, and should be made use of. Instead of using forceps to curtail the sufferings of labor, we had better consider the very great efficacy of morphia, chloroform, and chloral.

As an isolated indication, it is doubtful whether pain ever warrants forceps use, nor would such cases necessarily end with less suffering after immediate delivery.

It is a common occurrence for women to demand that the accoucheur shall do something to help the physiological phenomena of labor. Dr. Robert Barnes denounced this as an old and bad practice.

Anesthesia is the best treatment for cases of excessive nervousness and emotionalism in labor, not the forceps, as has been advised. Chloroform during the pains often makes labor of this sort more normal.

The nervous element is often misleading; it is not always the patient who is most noisy who suffers most, nor is it safe to be influenced by her cries for more chloroform or the forceps.

The loss of time by an accoucheur is not to be accepted as a warrant for resorting to instrumental delivery. We fear in these times of go-a-head-ativeness this is a common source of abuse.

The forceps is a life-saving instrument for both mother and child, whether we are dealing with the first or second stage of labor, provided they are used judiciously and skillfully.

There is much difficulty in obtaining exact statistics as to the frequency with which the forceps is used. The figures vary with the country and with the operator. Ploss's tables show a wide difference in frequency of application in German, Swiss and Russian maternities. In an aggregate of 33,054 labors, the forceps was applied 1,975 times, or one in thirty applications.

Ploss's tables for England make the least number of applications for any operator, as one in 3,878. The operator with the greatest relative number was one in 7.8. Jacquemier's tables give 20,517 labors and ninety-six forceps cases, or one in 214.

These tables embrace a period from 1792 to 1862. Those were the days of the swaddling infancy of obstetric knowledge, when method was just inaugurated by Baudelocque. Still they are useful in the light of history, in setting forth the wide range of opinion as to the frequent use of the forceps in those days.

Galabin says no one would now recommend so sparingly a use of forceps as one in 200. He says that the statistics of St. Thomas's Lying-in Charity appear to show that a forceps rate of one in sixteen to one in eighteen deliveries does not endanger the mothers; but wider statistics are desired.

The Maternity Hospital, Philadelphia, shows the number of forceps applications in 1886 to have been about one in eight.

The Columbia Hospital, Washington, D. C., has had an average of about one in twenty. At the Maryland Maternity Hospital, Baltimore, we have used forceps 142 times in 1,495 deliveries, or one in 9.4.

The number of forceps applications at Vienna Lying in Asylum during the past year was two per cent.

In my private practice, including consultation work, the average has been one in 10.5. I have found by inquiry of many practitioners that their average in private practice ranges from one in four deliveries to one in twenty-five.

It is to be deprecated that there is not a greater tendency to specialism in obstetrics. No man, ignorant of the mechanism of labor, and who does not know the rules for using the forceps, should ever undertake a high forceps operation. That clumsy, untutored and unskilled hands do harm with the forceps even at the lower strait cannot be denied. As long as this unfitness and want of training for obstetric operations exists, it will be humane to withhold our advocacy of a still more frequent use of forceps.

Nature has been defined as "the good will of God expressed in facts." We should crave her facts and imitate her. We will be adverse to substituting art for nature, so long as man is inferior to his Maker, except we act because of danger to mother or child.

We will not detain you with the enumeration of the many accidents and injuries which befall the mother and child at the hands of the forceps. Suffice it to say, they embrace a large part of every recent work on gynecology.

We sadly need statistics to settle the wide differences among obstetric operators with the forceps as to the frequency with which they may be used. Our judgment is that the average of one in fifteen or sixteen applications of forceps, as suggested by Dr. Galabin, is a fair one. The pendulum, having properly left the position of too great infrequency, has gone to an unwarranted extreme in point of frequency.

The obstetrician would do well to be found in the good company of the physician in the prevention of disease, the surgeon in his conservatism, and in line with the modern sanitarian.

Dr. Joseph Price, Philadelphia.—For years I condemned the use of forceps in unqualified language. During my hospital experience I singled out 300 cases of labor, in twenty-five of which forceps had been used in previous labors. In none of the twenty-five cases could I find the slightest indication for the use of forceps. I know that in skilled, trained hands it is a valuable instrument. I am glad the author has advocated the Tarnier forceps, and has called attention to the Taylor narrow-blade forceps. In one case—a very ugly patient—in five successive labors evisceration had been performed. She finally agreed to submit to induced labor, and thereafter three living children were delivered with the traction-rod and Sims' forceps. The Sims forceps have a great advantage in the greater diameter of the blade. I have gone to three series of 100 cases without taking my forceps out of the bag. In a discussion on the use and abuse of the forceps in our Obstetrical Society, one man admitted that he could not afford to attend poor women for five or ten dollars and not use forceps. It was five or six months before he came back to the Society, so thoroughly was he rebuked.

Dr. Franklin Townsend, Albany.—It is important to bear in mind that in labor the first stage is governed by the sympathetic nervous system; in the second stage, by the cerebro-spinal system. I do not believe the second stage should be allowed to continue longer than four hours. The exhaustion due to the strain on the cerebro-spinal nervous system is such as to warrant a speedy termination of labor.

### THE TECHNIQUE OF VAGINAL HYSTERECTOMY.

By Dr. James H. Etheridge, of Chicago.

### AN UNUSUAL CASE OF SUBSEROUS UTERINE FIBROID.

By Hampton E. Hill, of Saco, Maine.—Miss U. H., American; age, 40 years; height, four feet eleven inches; much emaciated; abdominal wall and lower extremities edematous. The ribs projected outwards; the tumor was very large for so small a woman.

The abdomen had a very firm feeling, the uterus was about the normal size, and slightly movable. There was a soft, fluctuating mass to the left of the uterus, and to the right a firmer feeling mass by vaginal examination. The general impression given by the examination led to the diagnosis of an ovarian fibro-cyst. The operation was made on January 3, 1888, with the assistance of Drs. J. E. Kimball and F. E. Maxcey, of Saco, Me., and Dr. M. C. Lathrop, of Dover, N. H. All aseptic and antiseptic precautions were observed. Upon making the abdominal incision, the tissues were very vascular. When the tumor was exposed, it was found to be a solid fibroid. The tumor was firmly adherent to most of the abdominal wall, and the adhesions were a network of blood-vessels. The hemorrhage was unusually severe. Twelve pairs of hemostatic forceps were in use before the operation was fairly begun; then the vessels were secured by ligation and torsion as fast as they were divided. Many ligatures were left on the abdominal wall.

The omentum was adherent in a vascular mass over the upper part of the tumor. The pedicle was secured by passing an ecraseur chain underneath the tumor and around the pedicle, and the tumor cut away from the pedicle. Then the lower end of the tumor was raised and drawn downward, a broad mass of vessels running into the back of the tumor from the mesentery was next secured by long clamp forceps, and the tumor divided from it. It was then removed from the abdomen, and the mesenteric vessels secured in three ligatures.

The pedicle was ligated with strong silk. A cyst of the left ovary, the size of an orange, was then tied and cut off, the abdomen cleansed with hot water, a drainage tube left in, and the wound closed, dressed, and the patient placed in bed, in a collapsed condition. Brandy had been used freely under the skin during the operation. Drainage was very free during five days. Tube removed in six days. There was an accumulation of fluid afterwards. Forty ounces were removed on the eighteenth day, after which the patient improved rapidly, and made a good recovery. The tumor measured in length sixteen inches; in width, fifteen inches; in thickness, ten and one-half inches. The weight was forty-seven pounds. The abdominal incision was extended from near the pubes to the ensiform cartilage.

Dr. Edward J. Ill, of Newark, N. J., read a paper on

### DESMOID (FIBROID) TUMORS OF THE ABDOMINAL WALLS.

He cited two cases, in one of which one hundred square centimetres of peritoneum was removed. In both cases the peritoneal cavity was opened. One case was operated upon by the excellent method of Saenger. Both cases recovered. The writer then spoke at length on the anatomy, pathology, etiology, symptomatology, diagnosis, and treatment of these rare forms of tumors. He drew especial attention to the symptoms produced by the contraction of the abdominal muscles on the tumors. He dwelt at length upon the differential diagnosis between this form of tumor and enlarged spleen, enlarged kidney, subcutaneous or subperitoneal tumors, uterine fibroids, solid tumors of the ovary and round ligament, adherent displaced kidneys, cyst of the urachus, dermoids of the abdominal walls, etc.

The prognosis of true dermoid is good. He has collected twelve cases of this form of tumor occurring in the United States. Ten of these occurred in females between the ages of twenty-three and forty-one years. In five of these cases, the origin of the tumor was given as the fascia transversalis. In six cases, the tumor was so closely adherent to the peritoneum that it became necessary to open the peritoneal cavity in removing them. In two of these, excision of a large portion of the peritoneum became a necessity.

Dr. Charles A. L. Reed, of Cincinnati, presented a paper on

FIBROID TUMORS OF THE ABDOMINAL WALL.

He commenced by referring to the dearth of American and English literature on tumors of this character. After accumulating his data, however, it became apparent that, to dispose of the entire topic, it would be necessary to discuss (*a*) fibromata, (*b*) lipomata, (*c*) myxo-lipomata, (*d*) cysto-carcinomata, (*e*) neuromata, and (*f*) hydatid cysts. A discussion of these sub-topics, however cursory, would result in the production of an essay too voluminous for this occasion. As a consequence, and in consideration of the fact that his own observations had been limited to five cases of tumor of the abdominal wall, all of them fibroids, he determined it expedient to restrict his paper to a discussion of that variety. He considered the subject under the following heads: history, etiology, pathological anatomy, symptoms, development, influence of pregnancy on the nutrition of these growths, duration, prognosis, diagnosis, and treatment. He presented the subject of treatment under three heads—exploratory incision, total extirpation, and electrolosis—reporting his five cases in detail. The conclusions to which he had been forced by a study of fibroids of the abdominal wall, and by his experience in their treatment, were as follows: *First*, fibroids of the abdominal wall, however small, benign or indolent, are liable to take on active growth; *second*, when small, their anatomical relations are unimportant and their extirpation can be accomplished without serious risk to the patient, and should be advised; *third*, when permitted to grow large, their anatomical relations become very important and their extirpation can be accomplished but with hazard to the patient; *fourth*, when in cases of large tumor, extirpation should be undertaken only where the exploratory incision demonstrated its feasibility; *fifth*, there should be no hesitancy in abandoning an operation when exploratory incision has shown it to be dangerous, because the primary incision may be of benefit to the tumor; *sixth*, in cases of manifestly deep-seated tumors with extensive peritoneal attachment, electro-puncture should be tried before extirpation is undertaken.

Dr. George H. Rohé, of Baltimore, by invitation, read a paper on

DISEASES OF THE SKIN ASSOCIATED WITH SEXUAL DISORDERS IN THE FEMALE.

[As this paper will be published in full in the November number of this journal, an abstract of it is omitted.—Ed. Buff. Med. and Surg. Jour.]

DISCUSSION OF EXTRA-UTERINE PREGNANCY—ITS PATHOLOGY.

By Dr. Franklin Townsend, Albany, N. Y.—Much of interest has been already published upon the subject of extra-uterine pregnancy, both of a theoretical as well as of a practical nature, but reference to results from broad studies, in comparative anatomy, is but scanty. In tracing out pathological conditions, we should of necessity refer to histology and physiology, for without these scientific aids to our study, we lose sight of the finer shades of beginning morbid processes from absolutely normal ones. We should begin our study of the pathology of ectopic gestation by comprehending the physiological functions of the organs most intimately involved. The pathological processes following may then, I think, be more distinctly and definitely traced. Most authorities are agreed that the functions of the Fallopian tubes are, first, to transmit the ovum from the ovary to the uterus, and, second, to permit the passage of the spermatozoa from the uterus in the direction of the ovary. There are a few, and the best of authorities, who differ from this view.

Physiology of Impregnation of the Ovum.—The seat of contact between ovum and spermatozoon has not yet been determined with absolute certainty. Many of the best authorities differ in their conclusions upon this subject. Coste's observations seem to prove that fecundation is almost always effected, either upon the ovary or in the part of the tube nearest the fimbriated extremity, inasmuch as he maintains that the ovule spoils very quickly when it enters the tube without previous fecundation. Regarding the functions of the tube and ovaries, Mr. Tait has proven, conclusively to my mind, that ovulation can and does take place before, during, and even after menstruation ceases; also, that the changes in the ovary at puberty are simply vascular, and that those in the tubes at this period are vascular, muscular and epithelial, and that the change of greatest importance is in the functional movement of those accessory organs. Ovulation, then, and menstruation are necessarily coincident. If, then, ovulation continues intermenstrually when the tubes are quiescent, what becomes of the ovum when the ovisac ruptures? Mr. Tait says: "I believe that the ovum falls into and perishes in the peritoneal cavity in by far the greater number of cases, and that the passage of it into the uterus occurs only in a small minority of the ova produced."

Accepting, then, the views of the majority of the authorities, that fecundation usually takes place either in the tubes or on the surface of the ovary, or even in the Graafian follicle, or possibly, as has been intimated by Parry, in the peritoneal cavity, and granting the admirable stand taken by Tait, it seems to me that, first, fecundation of the ovum takes place more frequently than is supposed. Second, that this being the fact, many sterile women, that is objectively sterile, who never complain of pain or ache, who ovulate and menstruate with greatest nicety and regularity, and whose general health is perfect, may frequently have fecundated ova, which, like non-fecundated ova, may drop into the peritoneal cavity and perish, because the soil there is unpropitious for their development. Third, that occasionally but rarely, I will admit, this same peritoneal soil, if I may be permitted to use such a term, does present a favorable site for the development of the fecundated ovum, and what is called "primary abdominal pregnancy" results. Fourth, this propitious site may be due to the old peritoneal inflammatory troubles, which may be so slight, indeed, as to have never given rise to suspicion of their existence. Such nesting spots in the peritoneum for the development of the young fecundated ovum, though occurring not so frequently as those inflammatory changes in the tubes, causing desquamation of the ciliated epithelium, and thereby tubal pregnancy, as Mr. Tait so ably advocates, are, nevertheless, a factor of causation of the so-called "primary abdominal pregnancy."

From the physiological proofs, as already cited, I am convinced that extra-uterine fetation can and does occur either in the Fallopian tubes, (by far the most frequent form,) in the ovary or upon it, and even in the peritoneal cavity, and I must truthfully say that, in the study of any given case of misplaced conception, one of the most perplexing questions to decide is as to which class it properly belongs: whether tubal, ovarian, or abdominal. By far the most frequent form is tubal ectopic gestation, ascribed usually to a number of causes, as catarrh of the mucous membrane, flexions of the tubes, dilatations with hernial pouches, or constriction from inflammatory changes. Naturally, the pathological changes taking place will vary according to the duration and behavior of the pregnancy. As the growth of the ovum continues, the mucous membrane of the tube thickens, the tubes themselves gradually distend, the villi enter into the mucous membrane, and, according to Bandl, "the two poles of the decidua-like covering are closed, though sometimes the uterine end remains open and in continuity with the mucous membrane of the tube and the decidua of the uterine cavity." The vascularity of the vessels of the tubes and those of the broad ligament in which they lie is greatly increased, the muscular fibres of the tube, enlarging at first, subsequently become markedly thin by stretching from the continued and increasing pressure, due to the growth of the ovum which finally ruptures the tube, usually between the second and third months. As a result of such tubal ruptures, the placenta is frequently lacerated, and the hemorrhage excessive, which pours into the peritoneal cavity, death being frequently due to shock, hemorrhage, or, if not from either of these, purulent peritonitis is apt to develop. Associated with the rupture in the wall of the tube may be that of the ovum, with the escape of the fetus into the peritoneal cavity, or it may be that the ovum remains whole, and in such condition falls into the abdominal cavity. Fatal as this form of ectopic gestation usually is, recovery may occur in case of premature death of the fetus before the tube gives way, and even after rupture has taken place recovery is possible, owing to the formation of inflammatory false membranes around the embryo of the entire ovum.

Ovarian Pregnancy.—So long ago as the latter part of the seventeenth century, St. Maurice demonstrated a case of ovarian pregnancy. A number of cases of this very rare condition are now on record.

Rupture of the sac takes place early, though when the sac walls are reënforced by inflammatory adhesions to the peritoneal coverings of adjacent viscera, gestation at full term may be reached.

Abdominal Pregnancy.—As has been shown, fecundated ova frequently drop into the abdominal cavity and perish. Occasionally it happens that their growth may continue for an indefinite period. The pathological changes occurring in this form of primary abdominal pregnancy must be distinguished from those that take place in what is termed "secondary." In the one instance we have so minute, soft, fragile and delicate a corpuscle deposited in the peritoneal cavity, that one could not well imagine any grave inflammatory results accruing from its immediate presence. This being the case, then, the contiguous abdominal organs will not be injured by its ulterior development. On the other hand, in the secondary form of intra-peritoneal pregnancy, we have a voluminous product of conception suddenly thrust upon the peritoneum, accompanied by large quantities of blood. Here the ovum acts the part of a foreign body, soon determining an acute inflammatory process about it which possibly may form a cyst-wall made up almost wholly of plastic lymph, which completely isolates it from the rest of the peritoneal cavity. If the fetal cyst ruptures, and the contents escape from the amniotic cavity into the midst of the intestinal mass, a renewal of the inflammation occurs and the cyst just described forms around it. As a rule, the fetus perishes at or soon after the time of rupture. With the death of the child, it may be converted into a lithopedion; or, through the blood supply of the connective tissue, it may be preserved for years in its soft integrity.

In all forms of ectopic gestation, the connection between the ovum and the abnormal surface upon which it is engrafted is established by a vital adhesion between the chorionic villi and the tissues with which they come in contact.

The Uterus in Ectopic Gestation.—From researches made by Clark, Oldham, Virchow and others, it would seem fair to conclude that the uterus is enlarged even in the early stages of ectopic gestation. A decidua forms in its cavity, which is usually expelled during the early stages of gestation en masse, with pain and symptoms of abortion, or it may be discharged in shreds and pieces without symptom.

### EXTRA-UTERINE PREGNANCY: ITS DIAGNOSIS.

By Dr. Joseph Price, of Philadelphia.—For the sake of brevity, all allusions to the literature of the subject have been omitted and the facts generalized. The literature of extra-uterine pregnancy is scattered through a vast number of periodicals, and consists for the most part of descriptions of cases occurring in the practice of the writers. The most valuable works are those of Mr. Lawson Tait and Dr. John S. Parry. Mr. Tait is undoubtedly correct in his proposition that all extra-uterine pregnancies are primarily tubal, and that all the so-called varieties depend upon the location of the ovum in the tube and the location of the point of rupture of the tube. The subject may be divided into three classes: *first*, extra-uterine pregnancy before the rupture of the tube; *second*, at the time of rupture of the tube; and *third*, after the rupture of the tube. Rupture of the tube is not synonymous with rupture of the fetal sac, though they generally occur at the same time. The diagnosis of extra-uterine pregnancy before the rupture of the tube is rarely made, and when it is made is of necessity not positive, because the same set of symptoms may arise from a number of pathological conditions. The symptoms they present are as follows: first, partial or complete cessation of menstruation for one or more periods, generally accompanied by other rational symptoms of pregnancy; second, pain which is peculiar, being generally severe, paroxysmal, and long-continued; a sickening pelvic pain which is neither cramp-like nor colicky. These pains, probably caused by the distention of the tube, are like to subside for a time, only to return. Third, the appearance of uterine hemorrhage, which is again peculiar in that it is usually irregular both as to time and quantity; generally lighter in color than the normal discharge and containing shreds of tissue, which are portions of the decidua vera. The general condition of the vagina and cervix may or may not correspond to normal pregnancy. The uterus is generally enlarged and pushed out of place by a tender or exceedingly painful cystic mass, occupying the position of one or the other of the tubes and freely movable. A differential diagnosis is extremely uncertain. Care must be taken not to hastily exclude pregnancy because of the apparent return of the catamenia, nor to conclude that miscarriage has taken place on account of the presence of tissue in the discharge. When the fetus and placenta die before the rupture of the tube, the difficulty of diagnosis is practically insurmountable. These symptoms continue as the pregnancy advances. The tumor, as it increases in size, causes additional symptoms by pressure on the bladder and rectum. Rupture takes place almost always between the eighth and fourteenth week of pregnancy; in a majority of cases before the twelfth week. Now the symptoms vary somewhat, according to point of rupture in the tube, whether into the peritoneal cavity or below it. If into the peritoneal cavity, as it is prone to do in a large majority of cases, the symptoms suddenly become most alarming. The patient is seized with agonizing pelvic pain, shows all the symptoms of internal hemorrhage and shock, goes into syncope, collapse and death. Dr. Formad, of Philadelphia, states that within a very short period he had found in his post-mortem work eighteen deaths due to ruptured tubal pregnancy. These deaths all occurred before the twelfth week of pregnancy. Where death does not immediately supervene, the recovery from shock is gradual; uterine hemorrhage generally occurs, symptoms of peritonitis make their appearance, and the patient slowly recovers only to undergo another attack of the same kind. Physical examination now may or may not present characteristic lesions. If the patient survives thus far, the symptoms of purulent peritonitis or septicemia set in, and finally death relieves the suffering woman. When rupture occurs below the peritoneum, the symptoms are rarely so severe and may indeed be scarcely noticed by the patient. The hemorrhage is rarely or never fatal at the time of rupture.

Here, again, recurrent attacks mark the progress of the case, and the ultimate outcome depends on whether the fetal sac is ruptured or not. Examination of this point will reveal a sensation of bogginess and distention in the pelvis. The symptoms of peritonitis are wanting generally. If the fetal sac has ruptured, the fetus dies, and if the condition is not recognized and relieved by operative measures, the patient goes into a state of chronic invalidism, though sometimes they recover fair health and comfort. In a small minority of cases, the fetal sac is not ruptured, and now the progress of gestation is similar to the normal until full term, providing a secondary rupture into the peritoneal cavity does not occur. After quickening, the doubts of pregnancy are settled and the question is the location of the fetus, whether within or without the uterus. The severe, paroxysmal pelvic pains may be very infrequent or cease altogether. The mammary changes are as in normal pregnancy. As the fetal sac enlarges, it produces unusually distressing pressure-symptoms on rectum, bladder, and blood-vessels. Physical examination is the only mode of determining the diagnosis. The fetal sac is generally less movable than the gravid uterus. Vaginal examination shows the uterus enlarged, but not in proportion to the duration of pregnancy, generally displaced to one side, in front or beneath the tumor. If the patient carries the extra-uterine gestation to term, spurious labor will take place. It is accompanied by metrorrhagia. After this spurious labor, the fetus dies and is disposed of in a variety of ways by nature.

### EXTRA-UTERINE PREGNANCY: ITS TREATMENT.

By Dr. E. E. Montgomery, of Philadelphia.—In the discussion of extra-uterine pregnancy Dr. Montgomery said, that when asked to take part, he chose the subject of the surgical treatment. While his subsequent study had not induced him to depreciate the value of surgery, it had led him to appreciate more highly than he had before done the possibilities of treatment by electricity.

Treatment must depend upon the form with which we are confronted.

Electricity affords an agent which can be relied upon for the destruction of fetal life. I am aware that its efficacy is questioned, but we cannot accept the dicta of men who are ignorant of the manner in which it is used, or of those who condemn it without a trial. An agent which is capable of destroying the life of mice and insects by passing a current through a vessel of water in which they are placed, should be effective in destroying life when brought in close contact with the fetal envelope through vagina or rectum, as may be most convenient. Its method of action is not electrolytic; the proper method of use is not by puncture. Those doubting its efficacy, question the diagnosis; but it is improbable that all of the cases quoted are cases of mistaken diagnosis. An agent that will dispel conditions affording the subjective and objective symptoms of ectopic gestation is worthy of further consideration.

In conclusion, he recommended the following plans of procedure:

1. In every form of ectopic gestation, prior to the fourth month, the destruction of life by electricity (faradic current).

2. Between the fourth and sixth months, destruction of life by electricity, and some weeks later laparatomy.

3. In rupture, immediate laparatomy, with removal of sac, contents, and the effused blood.

4. In cases that have passed the sixth month, wait until viability is well established, and perform laparatomy, observing every precaution that separation of the placenta does not occur, close the sac above, and drain through the vagina.

5. In case of death of fetus, it should be removed by laparatomy a few weeks later.

6. Where the fetus has become macerated and abscess has formed, its sinus should be enlarged and the fetal residue removed.

EXTRA-UTERINE PREGNANCY: ITS TREATMENT (CONTINUED).

By Dr. A. Vander Veer, Albany, N. Y.—Much of the literature upon ectopic gestation is very vague. The monograph of Sir Spencer Wells on "Abdominal Tumors" is especially valuable for its clear history of cases, and the concise, yet minute, description of the surgical devices employed.

The abdominal surgeon needs to be perfectly familiar with the points of diagnosis, especially in the early stages.

There is a class of cases in which a diagnosis can be made before rupture, and by the tenth week. Another class gives rise to no symptoms other than those of normal pregnancy before the rupture takes place. Witness the thirty-five cases of Tait and the eighteen cases found post-mortem by Formad, of Philadelphia. The first class are such patients as have been under the eye of the gynecologist, perhaps, for years. He will be conversant with the pelvic conditions and relations. In such cases, there will be the onset of menstrual disorders altogether new, severe recurrent attacks of pelvic pain, mammary and gastric disturbance, perhaps the expulsion of shreds of membrane. These symptoms, taken with the "wine cast" vaginal hue, are valuable in many cases as early as the second month. Then, with the uterus displaced, laterally or forward, a soft cervix, patulous os, uterus enlarged, and a new growth behind or at one side, globular, and semi-fluctuant, gives a strong presumption of ectopic gestation. The use of electricity as a feticidal agent is largely an American method of treatment. The strongest objection to its use seems to be that untoward results follow the operation. The operation for the removal of the product of an ectopic gestation before rupture has taken place is to the surgeon an ideal method. There remains no foreign body to be disposed of, and no serious after-effects are to be feared. Veit records seven successful cases with no failures. When the symptoms show that rupture has taken place, there can be no doubt that an immediate abdominal section is the only proper thing to do.

When ectopic gestation goes safely beyond the fourth month, danger of rupture is small.

The general tendency of operators is, then, to await spurious labor. Shall we then try to remove a living child, or wait until the placental circulation has ceased? The tendency of operators is growing more and more toward primary laparatomy. In view of our improved technique in doing the operation, I believe we are justified in urging primary laparatomy. In primary laparatomy, the placenta ought to be removed either by ligation or exsection.

DR. A. LAPTHORN SMITH, of Montreal, by invitation, read a paper on

SOME MINUTE BUT IMPORTANT DETAILS IN THE MANAGEMENT OF THE CONTINUOUS CURRENT IN GYNECOLOGY.

The following officers were elected for the ensuing year: *President*, DR. WM. H. TAYLOR, Cincinnati; *Vice-Presidents*, DR. E. E. MONTGOMERY, Philadelphia, and DR. J. HENRY CARSTENS, Detroit; *Secretary*, DR. WILLIAM WARREN POTTER, Buffalo; *Treasurer*, DR. X. O. WERDER, Pittsburg; *Executive Council*, DR. THOMAS OPIE, Baltimore; DR. CLINTON CUSHING, San Francisco; Dr. MELANCTHON STORRS, Hartford, and DR. BYRON STANTON, Cincinnati.

The next meeting will be held in Cincinnati, upon a date to be fixed by the Executive Council.

